# Bypass versus angio plasty in severe ischaemia of the leg - 2 (BASIL-2) trial: study protocol for a randomised controlled trial

**DOI:** 10.1186/s13063-015-1114-2

**Published:** 2016-01-06

**Authors:** Matthew A. Popplewell, Huw Davies, Hugh Jarrett, Gareth Bate, Margaret Grant, Smitaa Patel, Samir Mehta, Lazaros Andronis, Tracy Roberts, Jon Deeks, Andrew Bradbury, on Behalf of the BASIL-2 Trial Investigators

**Affiliations:** University Department of Vascular Surgery, Heart of England Foundation Trust, Netherwood House, Solihull Hospital, Lode Lane, Solihull, B91 2JL UK; Birmingham Clinical Trials Unit, Institute of Applied Health Research, College of Medical and Dental Sciences, Public Health Building, University of Birmingham, Birmingham, B15 2TT UK; Health Economics Unit, School of Health and Population Sciences, Public Health Building, University of Birmingham, Birmingham, B15 2TT UK

**Keywords:** Severe limb ischaemia, Critical limb ischaemia, Bypass surgery, Endovascular treatment, Angioplasty, Stent, Diabetes, Amputation

## Abstract

**Background:**

Severe limb ischaemia is defined by ischaemic rest/night pain, tissue loss, or both, secondary to arterial insufficiency and is increasingly caused by infra-popliteal (below the knee) disease, mainly as a result of the increasing worldwide prevalence of diabetes. Currently, it is unknown whether vein bypass surgery or the best endovascular treatment (angioplasty or stenting) represents the optimal revascularisation strategy in terms of amputation-free survival, overall survival, relief of symptoms, quality of life and cost-effective use of health care resources.

**Methods/Design:**

The Bypass vs. Angioplasty in Severe Ischaemia of the Leg - 2 Trial is a UK National Institute of Health Research, Health Technology Assessment funded, multi-centre randomised controlled trial that compares, at the point of clinical equipoise, the clinical and cost-effectiveness of a ‘vein bypass first’ and a ‘best endovascular treatment first’ revascularisation strategy for severe limb ischaemia due to infra-popliteal disease. The primary clinical outcome is amputation-free survival defined as the time to major (above the ankle) amputation of the trial limb or death from any cause. The primary outcome for the cost-effectiveness analysis is cost per quality-adjusted life year. Secondary outcomes include overall survival, quality of life, in-hospital mortality and morbidity, repeat and crossover interventions, healing of tissue loss and haemodynamic changes following revascularisation. Sample size is estimated at 600 patients. An economic evaluation will be conducted from the perspective of the National Health Service and comprise a ‘within-study’ analysis, based on prospectively collected trial data and a ‘model-based’ analysis, which will extrapolate and compare costs and effects beyond the study follow-up period.

**Discussion:**

The BASIL-2 trial is designed to be pragmatic and represent current practice within the United Kingdom. Patients with severe limb ischaemia can only be randomised into the trial where clinical equipose exists. The advent of hybrid operating procedures should not be a barrier to randomisation, should a patient require inflow correction prior to tibial revascularisation.

**Trial registration:**

ISRCTN:27728689 Date of registration: 12 May 2014.

## Background

As a result of smoking, diabetes, high blood pressure, high cholesterol, kidney failure, and old age, some people develop atherosclerosis (*aka* ‘hardening of the arteries) in their legs.

This atherosclerosis narrows and eventually blocks their arteries, thereby reducing the blood supply to their legs and feet: a condition termed ‘ischaemia’.

Advanced cases are known as ‘severe limb ischaemia’ and cause one or both of the following problems:

1. Injuries to the foot fail to heal, allowing infection to enter the tissues, resulting in the development of ulceration, and even gangrene.
2. Severe constant pain in the foot, which is often worse at night and disturbs the sleep.

In the developed world, the incidence of critical or severe limb ischaemia is estimated at 500 to 1,000 per million population [1].

The number of people affected by severe limb ischaemia is increasing worldwide as a result of the ageing population, the increase in diabetes, and the continuing high rates of smoking [2].

In people with severe limb ischaemia, if the blood supply to the leg is not restored (revascularisation), then the risks of amputation and death are high. In the UK, approximately 5,000 to 6,000 amputations are performed each year, the great majority for severe limb ischaemia [3].

1. In addition to best medical therapy (comprising anti-platelet and lipid modifying agents, optimal diabetic control, analgesia, and foot and wound care), which all patients should receive as indicated, severe limb ischaemia can be managed by means of the following:
2. Primary amputation, when the limb is beyond salvage and/or the patient is unfit/unwilling/unable to undergo revascularisation.
3. Surgical revascularisation, which is usually undertaken by means of a bypass fashioned using an autologous vein and/or endarterectomy.
4. Endovascular revascularisation, which is a ‘keyhole’ procedures performed through the groin under local anaesthetic and using balloons and stents to force/hold open the arteries.

The current evidence base underpinning the treatment of severe limb ischaemia is extremely poor with very few randomised clinical trials [4] and no available systematic reviews or meta-analysis.

The National Institute of Health and Care Excellence clinical guideline (CG) 147 on Peripheral Arterial Disease recommended that a randomised controlled trial be undertaken to answer the following question which BASIL-2 aims to answer: namely, ‘What is the clinical and cost effectiveness of a 'bypass surgery first' strategy compared with an 'angioplasty first' strategy for treating people with critical limb ischaemia caused by disease of the infra-geniculate (below the knee) arteries?’ [5]

### Aims

**Fig. 1:**
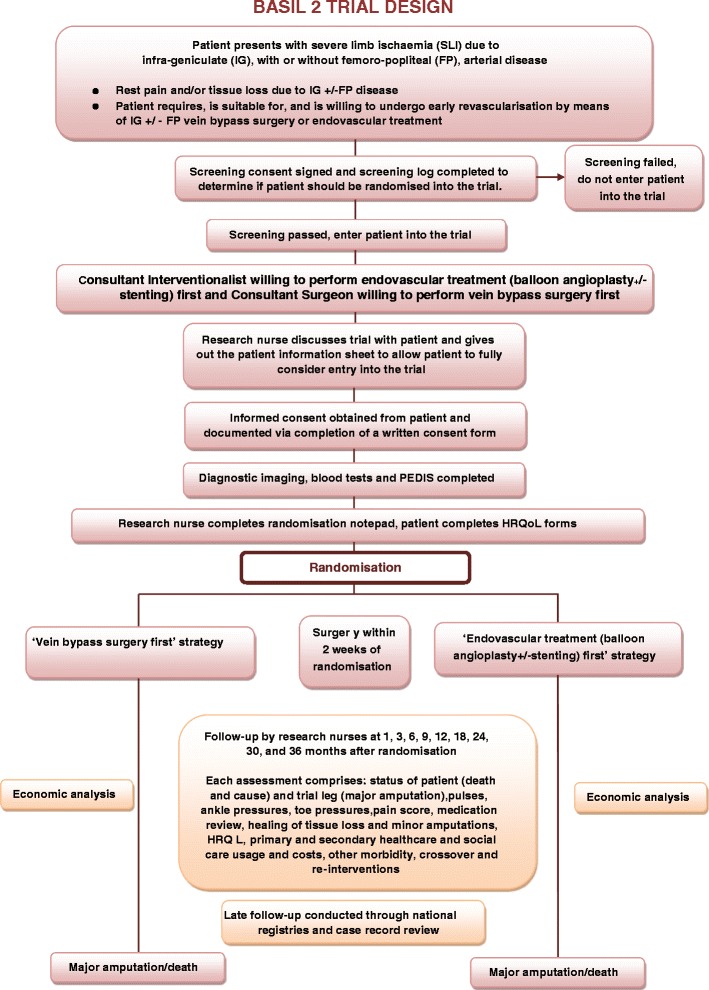
The BASIL-2 CONSORT diagram

The original BASIL-1 trial, on which BASIL-2 is based, randomised 452 patients with severe limb ischaemia, mainly due to femoro-popliteal disease (in the thigh), to either an angioplasty first or a bypass surgery first revascularisation strategy [4]. The Bypass vs. Angioplasty in Severe Ischaemia of the Leg - 2 (BASIL-2) Trial is a UK National Institute of Health Research, Health Technology Assessment funded, multi-centre randomised controlled trial (http://www.nets.nihr.ac.uk/projects/hta/123545) that will now compare, at the point of clinical equipoise, the clinical and cost-effectiveness of a ‘vein bypass first’ with a ‘best endovascular treatment first’ revascularisation strategy for severe limb ischaemia due to infra-popliteal (below the knee) disease (Fig. 1). BASIL-2 includes an internal pilot phase and economic analysis. The primary clinical outcome is amputation free survival defined as the time to major (above the ankle) amputation of the index (trial) limb or death from any cause, whichever comes first. The primary outcome for the cost-effectiveness analysis will be cost per quality-adjusted life year. Secondary outcome measures are shown in Table 1.

**Fig. 2:**
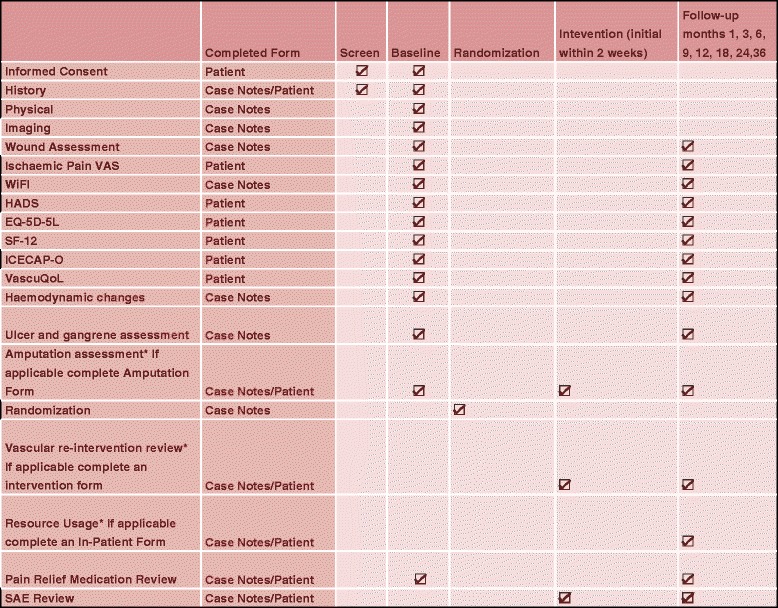
The BASIL-2 assessment schedule

**Table 1:** BASIL-2 secondary outcomes

Overall survival
In-hospital and 30-day morbidity and mortality
Major adverse limb event – amputation (transtibial and above) or any major vascular re-intervention (thrombectomy, thrombolysis, balloon angioplasty, stenting or surgery)
Major adverse cardiovascular event – (severe limb ischaemia and amputation affecting the contralateral limb, acute coronary syndrome, or stroke)
Relief of ischaemic pain (visual analogue scale and medication usage)
Psychological morbidity (Hospital Anxiety and Deprivation Score)
Quality of life using generic (European Quality of life 5 level questionnaire, Short Form-12, ICEpop CAPability for older people and disease specific Vascular Quality of Life) tools
Re- and cross-over intervention rates
Healing of tissue loss (ulcers, gangrene) of presumed arterial aetiology as assessed by the Perfusion Extent Depth Infection Sensation [6] and Wound Ischaemia Foot Infection [7] scoring and classification systems
Extent and healing of minor (toe and forefoot) amputations (also using the above wound scoring systems)
Haemodynamic changes: absolute ankle and toe pressure, (ankle brachial pressure index and toe brachial pressure index).

## Methods

### Ethics

Ethical approval was gained via the National Research Ethics Service, West Midlands, UK (Coventry and Warwick) on 3 March 2014 (reference 14/WM/0057). Each patient will provide fully informed, written consent prior to randomisation.

### Eligibility

All patients with severe limb ischaemia referred to vascular units within participating NHS Trusts can be considered. To be eligible for randomisation, patients must have adequate inflow to support infra-popliteal intervention, an anticipated life expectancy greater than 6 months, capacity to consent, and be able to speak English where translation facilities are unavailable (Table 2). Previous intervention to the trial leg is not an exclusion criterion.

**Table 2:** BASIL-2 inclusion and exclusion criteria

Inclusion criteria	Exclusion criteria
Have severe limb ischaemia due to infra-popliteal, +/− femoropopliteal disease	Have an anticipated life expectancy of < 6 months
Be judged by responsible clinicians (consultant vascular surgeon, interventional radiologist, and diabetologists) working as part of a multi-disciplinary team to require early revascularisation in addition to best medical therapy, foot and wound care.	Are unable to provide consent due to incapacity
Have adequate inflow to support the randomised infra-popliteal intervention (if not, patients can be randomised to have their allocated infra-popliteal intervention at the same time or after the inflow procedure).	Are a non-English speaker where translation services are inadequate to provide informed consent
Be judged suitable for both vein bypass and best endovascular treatment following diagnostic imaging and a formal documented multi-disciplinary team meeting.	Are judged unsuitable for either revascularisation strategy by the responsible clinician
	Tissue loss considered to be primarily of venous aetiology

Potentially eligible patients will be discussed at a formally minuted multi-disciplinary meeting comprising vascular surgeons and interventional radiologists. If the patient is deemed suitable for both treatment arms and there is clinical equipoise as to which revascularisation strategy is most appropriate, they will be invited to enter the trial.

### Randomisation

Blinding of the clinician and patient in this trial is not possible due to the completely different natures of the treatments allocated.

Randomisation is provided by a web-based third party service with a telephone option if required. The first 30 patients shall be allocated randomly, subsequently four out of every five patients are minimised, with 1 in 5 patients randomly allocated using random block without regular spacing. The following minimisation variables will be used:

- **age** (≤60, 61 to 70, 71 to 80, > 80 years),
- **sex** (male or female),
- diabetes mellitus and chronic kidney disease* (diabetes mellitus, chronic kidney disease, both or neither, where chronic kidney disease is defined as stage 3 or worse, based on estimated glomerular filtration rate of < 60 (ml/min/1.73 m^2^), and
- **severity of clinical presentation** (rest/night pain only, tissue loss only, or both).

### Baseline assessment

After randomisation patients undergo a baseline assessment comprising the following:

1. Patient history including risk factors for vascular disease, co-morbidities, previous arterial interventions to either leg, amputations and coronary interventions.
2. Physical examination including assessment of functional status, peripheral pulses, ankle-brachial pressure index and toe-brachial pressure index.
3. Review of investigations including imaging and results of blood tests.
4. Foot assessment using the Wound Ischaemia Foot Infection (WIFI) [6] and Perfusion Extent Depth Ischaemia Sensation (PEDIS) [7].
5. Assessment of Quality of Life using the Visual Analogue Scale, European Quality of life 5 level score, Short Form-12, ICEpop Capability for Older people, Vascular Quality of life, and Hospital Anxiety and Depression Scale.

The allocated intervention should be performed within 14 days where clinically and logistically possible.

### Trial interventions

Vein bypass will be performed using standard anaesthetic and surgical techniques. The type of vein used, and the location of the proximal and distal anastomoses, will be recorded. Best endovascular treatment will usually be performed under local anaesthetic via the common femoral artery. Success of vein bypass and best endovascular treatment will be assessed by pulse status, completion angiography and haemodynamic measures such as ankle brachial pressure index.

### Follow-up

Outcomes will be recorded at 1 month after intervention and at 3, 6, 9, 12, 24, 30, and 36 months after randomisation (Fig. 2). Information collected will include further interventions (vascular or non-vascular), hospitalisations (for whatever reason), other health problems, clinical and haemodynamic status of trial and non-trial limbs, functional status and quality of life assessments. The recording of Adverse Events and Serious Adverse Events will conform to the Good Clinical Practice standards and the Research Governance Framework 2005 [8, 9]. After delivery of the randomised intervention, all subsequent interventions will be at the discretion of the clinical team in the patient’s best interests.

### Sample size calculation

Sample size allocation was calculated on a time-to-event analysis, undertaken at 2 years post randomisation. Recruitment is aiming to take place over 3 years with 20 % recruited in the first year and 40 % recruited in each subsequent year. Non-event rates for amputation-free survival are assumed to be 0.72, 0.62, 0.53, 0.47 and 0.35 at years 1 to 5 based on data from the original BASIL-1 trial [4]. Allowing for a conservative estimate of 10 % drop-out rate for the primary outcome, a trial of 600 patients will have 90 % power to detect a reduction in amputation-free survival of one third (HR = 0.66 equivalent to an absolute difference of 12 % in amputation free survival at Year 3) with a 5 % significance level.

### Pilot phase

Recruitment will be reviewed at the end of a 12 month pilot phase. The following are proposed as criteria on which to base a decision to discontinue the trial at that stage:

- Less than 2/3 of the centres recruiting.
- Fewer than 60 patients randomised in total.
- Less than 2/3 of centres recruiting fewer than two patients per month after month 4 onward.
- Less than 80 % of patients have received their allocated treatment.

At the end of the pilot phase, the completeness of the quality-of-life data will also be assessed. If completion rates are poor, then consideration will be given to discontinuing the Hospital Anxiety and Depression Scale and ICEpop CAPability for older people tools.

### Repeat and cross-over interventions

Repeat and cross-over interventions are permitted in either arm of the trial if the primary intervention is unsuccessful. After reviewing the data from the original BASIL trial [4], we estimate a 20 % re-intervention rate, which we believe is more likely to occur in the best endovascular treatment arm and within the first 12 months of the primary intervention. Data will be collected on all re-interventions. Re-intervention rates will be compared in a secondary analysis (the trial is powered at 90 % to detect a two-fold difference of 10 % versus 20 %).

### Subgroup analysis

Subgroup analysis will be used to detect treatment effects between pre-specified variables, focusing on effects on survival and repeated measures models (Table 3).

**Table 3:** BASIL-2 sub-group analysis

Rest pain versus tissue loss versus both
Diabetes mellitus
CKD
Haemodynamic measurements
Alternative endovascular options
Differences in resource usage and outcome between alternative endovascular options

### Economic evaluation

An economic evaluation will be undertaken to determine the relative cost-effectiveness of the ‘vein bypass first’ and ‘best endovascular first’ revascularisation strategies with a view to informing clinical decision-making within the NHS. The economic analysis will consist of two components: a ‘within-study’ analysis which will be based on study end-points and a ‘model-based’ analysis which will extrapolate and compare costs and effects likely to accrue beyond the study follow-up period. The analysis will be presented in terms of cost per additional quality-adjusted life year gained and cost per year of amputation-free survival. In line with existing recommendations, the base-case analysis will adopt a health care system (payer’s) perspective by considering costs incurred by the NHS and personal social services. Costs will be calculated on the basis of patient-level data related to (a) the procedures under assessment; (b) hospital stay and re-admissions, and (c) post-discharge use of National Health Service and social services. Procedure-related costs will be estimated in a separate micro-costing study. If plausible, additional analyses will be undertaken from a wider societal perspective. Quality of life will be derived from patients’ responses to the EuroQol EQ-5D-5 L [10] instrument and will be translated into preference-based quality of life indices using a UK-specific valuation set [11]. In addition to the trial-based evaluation, a model-based analysis will be conducted to consider the costs and benefits likely to accrue over a lifetime time horizon. Both deterministic and probabilistic sensitivity analyses will be undertaken to explore the robustness of the obtained results to sample variability and plausible variations in key assumptions and employed analytic methods [12].

### Interim analyses

Interim analyses will be performed on an annual basis. A Haybittle-Peto approach will be used where all interim analyses use a difference of three standard errors as a stopping guideline. These analyses will be reviewed by the independent data monitoring committee on an annual basis or more frequently if required.

Differences in the primary clinical outcome (amputation-free survival) will be assessed by comparing time from randomisation to major (above ankle) limb amputation or death from any cause (whichever occurs first) between the two arms. Primary analysis will use Kaplan-Meier plots and test the difference between two arms using the log-rank test. Data will be censored when individuals reach the end of follow-up or are lost to follow-up before incurring the primary outcome.

Further analysis of the primary outcome will involve the fitting of flexible parametric survival models to estimate both relative and absolute differences in hazard of the primary outcome to model the underlying differences in hazard and to allow for non-proportional hazards. The addition of covariates to the model, derived from the minimisation variables (age, sex, presence or absence of chronic kidney disease, diabetes and tissue loss) will allow baseline adjustments and the ability to test for further subgroup effects. The primary analysis will be performed on an intention to treat basis taking into consideration the first allocated method of revascularisation, regardless of cross-over or repeat interventions.

Secondary outcome measures that are based on continuous scales will be examined using a repeated measures multi-level model to examine the effects over time. Treatment effects will be reported from these measures at 1 and 12 months from randomisation and at the end of the study. Other outcomes will be measured using standard statistical methods, such as Fisher’s exact test for categorical data and log-rank for time to event data. All of these analyses will be performed using the intention to treat principle with 95 % confidence intervals with associated *p*-values.

## Discussion

The trial is designed to be pragmatic and representative of the ‘real world’ NHS management of severe limb ischaemia due to infra-popliteal disease. Many of these patients will have multi-level disease including ‘inflow’ or aorto-iliac disease, which may need to be corrected prior to randomisation. With the evolution of hybrid operating it would be permissible to perform an on-table inflow procedure (to popliteal, knee, level) at the same time as the randomised infra-popliteal intervention. Alternatively, an inflow procedure to popliteal (knee) level can be performed first and the patient then randomised if further infra-popliteal intervention is considered necessary.

Data from the original BASIL-1 trial [4] suggests that up to 25 % of patients may present with bilateral severe limb ischaemia. Bilateral simultaneous revascularisation is rare. It is usually clinically obvious which leg requires revascularisation first. Only one leg can be randomised in BASIL-2.

Patients can only be randomised if clinical equipoise exists. Local variations in practice may influence such decisions with some centres likely to favour an endovascular intervention strategy and others preferring surgical bypass. Current evidence does not support either an endovascular first or bypass first strategy and unless an obvious clinical reason is evident (such as lack of vein conduit, unsuitability for a surgical intervention due to comorbid state) all patients who are suitable for both arms could, theoretically be randomised into the trial.

## Trial status

The BASIL-2 trial is currently open for recruitment in multiple UK-based centres and has randomised more than 100 patients at the time of publication.

## Abbreviations

BASIL: Bypass versus Angioplasty in Severe Ischaemia of the Leg
CG: clinical guideline
EQ-5D-5 L: European Quality of Life-5 dimensions-5 levels
HR: Hazard Ratio
ICECAP-O: ICEpop CAPability measure for Older people
ISRCTN: International Standard Randomised Control Trial Number
NHS: National Health Service
PEDIS: Perfusion, Extent, Depth, Infection, Sensation foot score
SF-12: Short Form 12 QoL questionnaire
UK: United Kingdom
VascuQoL: Vascular QoL Questionnaire
WIFI: Wound Ischaemia Foot Infection score
